# Perceptions of Generational Conflict Among Three Age Groups in South Korea

**DOI:** 10.1093/geroni/igab046.3032

**Published:** 2021-12-17

**Authors:** Ahyoung Lee, Soondool Chung, Juhyun Kim

**Affiliations:** 1 EWHA Womans University, Seoul, Seoul-t'ukpyolsi, Republic of Korea; 2 Ewha Womans University, Ewha Womans University, Seoul-t'ukpyolsi, Republic of Korea; 3 Chungnam National University, Daejeon, Taejon-jikhalsi, Republic of Korea

## Abstract

After rapid industrialization during the past few decades, the gap between generations in South Korea has widened and the issue of generational conflict is being discussed as a social problem (Chung & Lim, 2018). The purpose of this study is to find out how each generation perceives generational conflict in the areas of family, politics, economy and social welfare, and culture. An online survey of 1,000 adults aged 20 and over was conducted nationwide in South Korea in January, 2021 with three age groups: the youngest group aged 20-39, mid-age group of 40- 64 and the oldest group of 65 and over. The questionnaire was created using the items developed by a previous research that used a Delphi technique (Chung, 2020). Participants answered how serious they perceive generational conflict in the dyadic relationship on 5-point Likert scales. Descriptive statistics were calculated, and t-tests have been performed to see the generational differences. Results show that the youngest group and the oldest group perceive the highest level of generational conflict each other in the areas of culture and politics. In cultural aspects, ‘use of slang among the same group’, ‘Ability to utilize digital devices’ were the items that had the highest level of conflict. In the political realm, progressive vs. conservative ideology was the area of the highest conflict. In addition, t-test results showed that the oldest group perceived generational conflict even deeper than the youngest group in the ‘economy and social welfare’ and cultural areas. Implications of these findings are discussed.

